# Pharmacists’ perceived barriers towards delivering their emergency roles during the COVID-19 pandemic and perceived policymakers’ responsibilities

**DOI:** 10.1186/s40545-020-00254-y

**Published:** 2020-08-16

**Authors:** Iman A. Basheti, Razan Nassar, Muna Barakat, Rajaa Alqudah, Rana Abu Farha, Tareq Muqatash, Samar Thiab, Bandana Saini

**Affiliations:** 1 Department of Clinical Pharmacy and Therapeutics, Applied Science Private University, Amman, Jordan; 2 Department of Clinical Pharmacy, Jordan University of Science and Technology, Amman, Jordan; 3 Department of Pharmaceutical Chemistry and Pharmacognosy, Applied Science Private University, Amman, Jordan; 4 College of Pharmacy, University of Sydney, Sydney, Australia

**Keywords:** Coronavirus, Pandemics, Pharmacy educators, Educational institutes, Faculty of Pharmacy, Pharmaceutical association, Jordan

## Abstract

**Rational:**

In March 2020, the World Health Organization (WHO) declared the coronavirus infectious disease as a pandemic referred to as COVID-19. As an essential service, community pharmacists have been enacting a key role in patient counseling and supply of essential medicines and protective equipment.

**Objectives:**

To investigate pharmacists’ perspectives of the role of educational institutes and professional pharmacy organizations in supporting them to take on roles during COVID-19 pandemic and to identify barriers to be able to support themselves and their patients.

**Methods:**

This descriptive mixed-method study was conducted via a cross-sectional online survey distributed to pharmacists/pharmacy students in Jordan during the COVID-19 outbreak (15–30 March 2020) using an online questionnaire, followed by an online focus group. Questionnaire items related to participants’ perspectives in being prepared for and supported in their roles during the COVID-19 pandemic, and items were tested for face validity. Data were descriptively analyzed using the Statistical Package for the Social Sciences and triangulated with focus group findings.

**Results:**

Considering that fear and anxiety are a consequence of mass social distancing/quarantine, study participants (*n* = 726, age = 26.9 (SD = 8.0) years, 71.9% females), reported needing training on mental healthcare to be able to support themselves and people during pandemics (90.2%). Most respondents agreed/strongly agreed (59.7%) with the statement around pharmacy educators/educational institutes having a key role in preparing pharmacists for practice during epidemics/pandemics and agreed that their faculties should add a course regarding pandemic preparedness in their curriculum (89.9%). Results were similar regarding roles for the pharmaceutical associations. Focus group findings (*n* = 7) mirrored the survey findings to a large extent.

**Conclusions:**

Most participants believed that pharmacy educators and pharmaceutical associations have a role in preparing them to deal with the COVID-19 pandemic through online educational workshops/webinars. Online education on mental healthcare is specifically needed.

## Introduction

In March 2020, the World Health Organization (WHO) declared the coronavirus infectious disease 2019 as a pandemic caused by the coronavirus strain identified and termed “severe acute respiratory syndrome coronavirus 2” or SARS CoV-2, colloquially referred to as COVID-19 [1, 2]. This pandemic first emerged in Wuhan, followed by a worldwide outbreak in many countries and regions in succession [1, 2]. COVID-19, an extremely contagious virus infection [3], which may manifest in symptoms of mild to moderate respiratory distress including shortness of breath and dry cough in addition to fever [4]. The current pandemic has been preceded in the past two decades by two major pandemics from two different betacoronaviruses, Severe Acute Respiratory Syndrome coronavirus (SARS-CoV) and Middle East Respiratory Syndrome coronavirus (MERS-CoV), resulting collectively in more than 10,000 cumulative cases, with fatality rates of 10% for SARS-CoV and 37% for MERS-CoV [5]. To date, COVID-19 has no proven treatment or vaccine as yet [5].

It is worth highlighting that public health experts have requested a serious response from health professionals to help in controlling such pandemic events [6]. This is vital in a time where healthcare systems are facing various challenges at all stages of the outbreak. For example, in the United State of America (USA), a lag in test kit provisions has been identified; in response, the Centers for Disease Control and Prevention (CDC) opened a website (http://www.coronavirus.gov) to help people decide whether they should seek testing or not [7]. The American Medical Association, one of the largest medical associations in the USA, called the emergency declaration “necessary to help ensure that America’s health system has sufficient resources” [7]. International Health Regulations (IHR) guided management for managing the coronavirus pandemic has been adopted in many countries, to ensure the readiness of the health care system for such emergencies [8]. Those regulations are issued for the prevention, detection, and responsiveness to serious incidences such as today’s incident-the coronavirus pandemic.

At the healthcare professional level, doctors and nurses are fighting an uphill battle in the frontline, as are community pharmacists [9]. Community pharmacists can and are contributing to the health system through essential medicines supply, health education, and health promotion activities, in addition to administering vaccines to patients (e.g., vaccine against the influenza, though there is no current vaccine to prevent COVID-19 infection) [10–12]. The International Pharmaceutical Federation (FIP) released a guideline on the 19th of March 2020 clarifying the required coronavirus information for pharmacists and the pharmacy workforce [13], acknowledging the responsibilities put upon the pharmacists in the control of the COVID-19 outbreak [13]. New roles and responsibilities included the stable supply of key medicines and preventative products, provision of information about the coronavirus pandemic, performing early detection of cases via patient questioning about signs and symptoms, appropriately referring patients, providing sufficient tailored staff training, providing psychological support to patients [14, 15], and implementing governmental and policymaker mandated procedures [16, 17]. Pharmacists were also expected to play a role in promoting a culture of empathy, by helping prevent the stigmatization of infected individuals based on ethnicity, population or nationality, and by broadcasting COVID-19 facts in their communities via various media [18].

While global organizations such as the FIP have developed current guiding principles for pharmacists, the reality for pharmacists on the frontline may be rather different with fear, anxiety, and uncertainty governing the perceptions about pandemic-related roles and with lofty expectations from policymakers to overcome this dilemma. There are no current studies exploring in-depth pharmacists’ perceived fear from delivering the roles expected of them and what support they perceive to receive from the key stakeholders such as local policymakers, pharmacy educators/educational institutes, and professional pharmacy bodies/organizations in preparing pharmacists to take on emergency management roles during epidemics/pandemics with the skills needed to support themselves and their patients emotionally.

The aim of this study, therefore, was to investigate pharmacists and pharmacy students’ perspectives of the role of pharmacy educators/educational institutes and the professional pharmacy organizations in supporting them to take on emergency management roles during pandemics, including the COVID-19 pandemic, and to identify and overcome certain barriers to be able to support themselves and their patients optimally. For this study, Jordanian pharmacy practice served as a case in context, though the issue may be universally applicable.

## Methods

### Study design and participants

This study was carried out over 2 weeks during March 2020, during the coronavirus outbreak and public quarantine in Jordan. A descriptive cross-sectional study design was used to assess pharmacists’ perspective of the role of the pharmacy educators/educational institutes in Jordan and the Jordan Pharmaceutical Association (JPA) in preparing future pharmacists to deal with epidemics/pandemics, with a close focus on the coronavirus pandemic. The JPA is the only official pharmacists’ syndicate in Jordan, established in 1957 according to the Pharmacy Law in the Hashemite Kingdom of Jordan; more than 15,000 pharmacists are affiliated with it, which is the total number of practicing pharmacists in Jordan as membership is mandatory, meaning that a pharmacist may not practice the profession unless he/she is affiliated with the association.

A questionnaire was developed with items based on the current information regarding the pandemic in order to meet the study objectives. To be eligible, participants needed to be licensed community or hospital pharmacists, pharmacy academics, or enrolled pharmacy students in Jordan. Participation in the study was voluntary, and no risk was posed to the participants. Ethics approval was gained from the Faculty of Pharmacy, Applied Science Private University. The research team considered the participant who completed the questionnaire and submitted his/her responses to have given informed consent for participation in this study.

The sampling strategy included running the Facebook on a special Facebook page designed for this study titled “*The Coronavirus Pandemic and Pharmacy*”*.* This Facebook page stayed active until the targeted sample size was reached. The page started with “All pharmacists and students receiving pharmacy education in faculties of pharmacy (i.e., universities and institutes providing accredited pharmacy degrees leading to registration) in Jordan are kindly requested to give us a portion of your valuable time to fill out the questionnaire below which aims to identify important recommendations to decision-makers based on our needs and viewpoints; It also assesses our awareness as pharmacists and hence our readiness to fight epidemics/pandemics with a focus on the coronavirus pandemic. Thank you in advance for your valuable contribution”.

### Survey development

Based on an extensive literature review [5, 7, 13, 19], a relevant pool of questions was generated from a variety of sources, in order to construct the first draft of the questionnaire. The research team decided to compose the questionnaire in English since this is the main medium of instruction used in pharmacy education by provider institutes in Jordan.

In order to ensure the face validity of the questionnaire, six independent academic experts in the areas of Pharmacy Practice and Education were chosen by the research team to evaluate the first draft. Through this process, the draft survey was sent via a link to the six experts independently, with certain assessment criteria sent to them to mark each item; (suitability of the wordings used, clarity of the questions, suitability of the content (i.e., presence of a logical link between each question and the study objectives), comprehension of the questions, consistency of layout and style, and feasibility of the questionnaire); a space for open-ended comments was also added. They also informed the research team whether each question was relevant for inclusion with respect to the study objectives or if it had no added value if included. Their comments were used to produce the final version of the questionnaire, and their recommendations were incorporated where suitable. The questions were then tabled and revised by the research team in order to assimilate concepts and to eliminate duplicates. All questionnaire items were also mapped back to the study objectives to ensure focus. Finally, the research team reviewed the questionnaire for online administration suitability (format, nesting, sequencing, graphic layout, and general clarity).

The final developed questionnaire consisted of three sections assessing several domains of interest. The *first section* included items to collect participants’ demographic data. The *second section* included items aimed at assessing participants’ perspective of the role of the pharmacy educators/educational institutes and the professional pharmacy organizations in preparing future pharmacists to deal with epidemics and pandemics, with a specific focus on the coronavirus pandemic. There were several detailed items, e.g., if faculties should add a course to cover information on epidemics/pandemics and the role of the pharmacist during such times, the need for online lecture and webinar provision for students and alumni, online educational workshop provision on the current coronavirus pandemic, and provision of online information resources discussing international and local needs and findings (e.g., a summary of local and international research outputs) on the coronavirus pandemic. The *third section* included items aimed at assessing participants’ perspectives of the role of the JPA in preparing future pharmacists to deal with epidemics and pandemics, again using the current coronavirus pandemic as a case in point. Here participants were asked if the JPA should be sending registered pharmacists awareness emails clarifying important issues regarding the coronavirus pandemic or should be providing online educational workshops, online training resources, or patient education materials for distribution. Participants were also asked if the JPA should have a role in monitoring the availability of the medications used in the management of the coronavirus infection in community pharmacies (e.g., to counter disease mongering via social media and resultant hoarding response of consumers), and lastly, if the faculties of pharmacy nationally and the JPA should join forces and produce one educational module for the management of the pandemic coronavirus.

The last two sections included 5 closed-ended questions, and the participants’ responses to these sections were conveyed using a Likert scale, i.e., “Strongly agree”, “Agree”, “Neutral”, “Disagree”, or “Strongly disagree”.

### Survey implementation

Participants were targeted through the social media mainly via Facebook (study specific page shared by the individual study researchers) and WhatsApp groups (individual researcher’s WhatsApp groups). In the beginning, eligible participants who indicated a willingness to participate in this study were able to open a link to see the study ethics committee approved information, and afterward, they were able to complete the different items in the questionnaire. The questionnaire was designed to take less than 10 min in peer review focus group.

### The online focus group meeting

After the online questionnaire part of the study was completed, an open invitation was extended to policymakers to attend an online focus group session to elicit further comments about the role of the faculties of pharmacy (pharmacy education providers) nationally and the JPA during the COVID-19 pandemic in preparing pharmacists for their role. The invitation was sent via WhatsApp to a convenience sample of policymakers identified by the research team. The aim of the online meeting was clearly explained, the timing was set, a link and password to the Zoom online meeting were provided, and names of the meeting facilitators (authors IB and ST) were on information sheets sent with the invite. A series of open-ended questions were prepared by the research team as a basis for focus group format. The questions were as follows: (1) What do you think the role of pharmacy educators nationally should be in preparing pharmacy students to be engaged in combating pandemics? (2) What do you think pharmacy educators should be doing nationally now to combat the coronavirus pandemic? (3) What do you think the JPA should be doing to combat the coronavirus pandemic? (4) How happy are you with the actions that have been taken by the ministry and different healthcare institutions in Jordan to combat the spread of the coronavirus pandemic? (5) Any other comments?. In preparing these questions, the comments of the experts about the online questionnaire were borne in mind and prompt to stimulate discussion for each question prepared for use by facilitators during the focus group discussion.

### Sample size

Considering the reported number of licensed pharmacists in Amman (15,045) [20], the sample size calculation was completed by using a margin of error of 5%, confidence level of 95%, and 50% response distribution. A sample size of at least 375 pharmacists was found to be needed.

### Statistical analysis

Following data collection, the survey responses were coded and entered into a customized database using the Statistical Package for the Social Sciences (SPSS), version 22.0 (IBM Corp., Armonk, NY, USA). Descriptive results were presented as means and standard deviations for continuous variables and percentages for qualitative variables. An independent sample *t* test was performed to the difference in perception scores between students and pharmacists. All tests were two-tailed. A *p* value of < 0.05 was considered statistically significant.

Data from the recording of the online focus group conversation were summarized thematically by transcribing the focus group discussion verbatim, coding relevant text, and coalescing these codes into meaningful themes in an inductive approach. The coding and thematic analysis were undertaken by several members of the research team, following the constant comparison method [21].

## Results

Study participants (*n* = 726; pharmacists *n* = 470, students *n* = 256) had a mean age of 26.9 (SD = 8.0) years, and 71.9% were females. Participants were alumni of the public (45.9%) and private (54.1%) universities. Participants’ education achievements ranged along bachelor in pharmacy degree (40.2%) and doctor in pharmacy degree (6.2%), while the others had higher studies (diploma, masters or a Doctor of Philosophy (Ph.D.) degrees) (18.3%). Most participants worked in community pharmacies (17.1%), hospital pharmacies (4.5%), or as academics (9.4%). The majority were still students or student trainees (61.7%). Most of the participants lived in Amman (69.4%), were still studying (50.8%), or had graduated within the previous 5 years (28.1%), and hence most of them reported 1 to 5 years of experience (78.8%). Participants reported not attending a continuous professional development workshop during the previous year (26.6%), while others attended one (22.5%) or two workshops (19%) only (Table 1).

**Table 1:** Demographic characteristics reported by study participants (*n* = 726)

Parameter	Mean (SD)	*n* (%)
Age (mean ± SD)	26.9 (SD = 8.0)	
Gender, *n* (%)		
Female		522 (71.9%)
Male		204 (28.1%)
University		
Private university		393 (54.1%)
Public university		333 (45.9%)
Educational level		
Student		256 (35.3%)
B. Pharm		292 (40.2%)
Pharm. D		45 (6.2%)
Higher studies		133 (18.3%)
Employment		
Community pharmacy		124 (17.1%)
Hospital pharmacist		33 (4.5%)
Pharmacy student/trainee		448 (61.7%)
Academic		68 (9.4%)
Others		105 (14.5%)
Living area		
Amman		504 (69.4%)
Others		211 (29.1%)
Missing data		11 (1.5%)
Graduation years		
I am still studying		369 (50.8%)
1–5 years ago		204 (28.1%)
6–15 years ago		102 (14.0%)
16–25 years ago		31 (4.3%)
More than 25 years ago		20 (2.8%)
Years of experience		
1–5 years		572 (78.8%)
6–10 years		82 (11.3%)
11–15 years		23 (3.2%)
16–20 years		17 (2.3%)
More than 20 years		32 (4.4%)
The number of attended educational workshop in the last year		
0		193 (26.6%)
1		163 (22.5%)
2		138 (19.0%)
3		83 (11.4%)
4 or more		149 (20.5%)

**Fig. 1:**
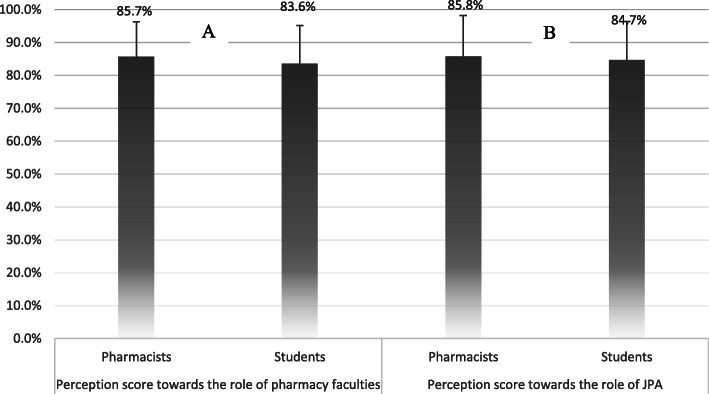
Differences between pharmacists (*n* = 470) and students’ (*n* = 256) perception scores regarding their perception towards the role of pharmacy faculties (**a**) (*p* = 0.013) and their perception towards the role of JPA (**b**) (*p* = 0.247) to deal with epidemics/pandemics and Coronavirus specifically

Participants’ opinions about their level of personal fear and anxieties about the coronavirus pandemic showed that 78.1% of them agreed/strongly agreed that working in a community pharmacy increases their concern about contracting the coronavirus infection. Significant difference between pharmacists (*n* = 470) and students’ (*n* = 256) scores regarding their perception towards the role of faculties of pharmacy to deal with epidemics/pandemics and coronavirus specifically (*p* = 0.013) was found, but not the JPA (*p* = 0.247) as shown in Fig. 1.

**Fig. 2:**
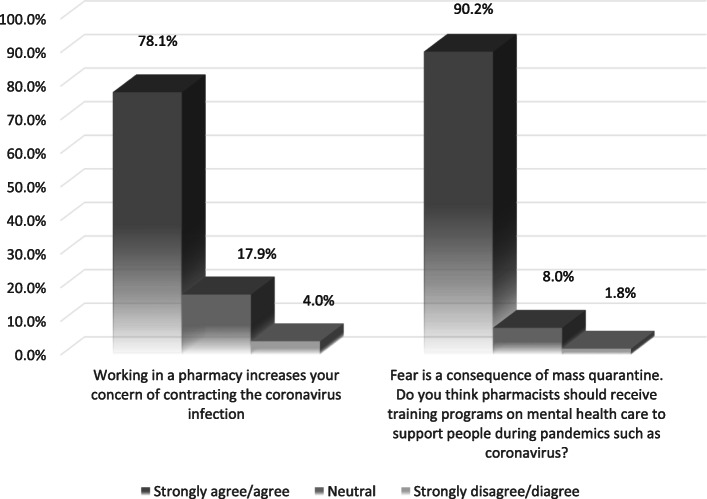
Participants (*n* = 726) perceived opinion regarding their fear from coronavirus pandemic

Considering that fear is a consequence of mass quarantine, a majority of participants agreed/strongly agreed (90.2%) that pharmacists should receive special training programs on mental healthcare and mental health first aid to be able to support people during pandemics such as the coronavirus pandemic (Fig. 2). Moreover, perceptions of participants regarding the role of the pharmacy education providers (university faculties or other institutes) showed that the majority agreed/strongly agreed (59.7%) with their faculty (e.g., the one they were studying in or were alumni of) having a role in preparing pharmacists to deal with any epidemic/pandemic. The majority agreed/strongly agreed that their faculty should add an epidemic/pandemic management course to their study plan (89.9%) and that their faculty should provide online lectures/webinars (86.4%) and online educational workshops (88.9%). The provision of online information resources such as summaries of current research studies on the pandemic coronavirus was also agreed/strongly agreed on by 92.7% of the study population (Table 2).

**Table 2:** Perceptions of participants (*n* = 726) regarding role of the faculties of pharmacy in preparing pharmacists for their responsibilities during epidemics/pandemics, and specifically the coronavirus pandemic

	Strongly disagree	Disagree	Neutral	Agree	Strongly agree
Your faculty has a role in preparing you to deal with any epidemics/pandemics	30 (4.1%)	84 (11.6%)	174 (24.0%)	238 (32.2%)	200 (27.5%)
Your faculty should add an epidemics/pandemics management course	1 (0.1%)	10 (1.4%)	62 (8.5%)	274 (37.7%)	379 (52.2%)
Your faculty should provide you with online lectures and webinars as a student or alumni	1 (0.1%)	12 (1.7%)	86 (11.8%)	280 (38.6%)	347 (47.8%)
Your faculty should provide online educational workshops on the pandemic coronavirus	3 (0.4%)	10 (1.4%)	68 (9.4%)	285 (39.3%)	360 (49.6%)
Your faculty should provide online information resources (e.g., summaries of current research studies) on the pandemic coronavirus	2 (0.3%)	6 (0.6%)	49 (6.7%)	259 (35.7%)	412 (56.7%)

Regarding pharmacists’ perceptions of the current role of the JPA in combating epidemics/pandemics and the coronavirus infection, the majority believed the JPA has a role in preparing pharmacists to deal with epidemics/pandemics such as the coronavirus pandemic (60.9%) and that the JPA should be sending them awareness emails (87.1%) and provide online educational workshops (86.4%) on the coronavirus pandemic. Many believed that the JPA should monitor the availability of the medications used in the management of the coronavirus in the pharmacies (89.2%) while 9.4% were not sure. The highest percentage of agreement reported in this section was the proportion of participants (90.4%) who reported that the pharmacy education providers (universities and institutes providing accredited pharmacy degrees leading to registration) and the JPA should join forces to produce one educational module for the management of the coronavirus pandemic (Table 3).

**Table 3:** Perceptions of study participants (*n* = 726) of the current role of the Jordan Pharmaceutical Association (JPA) in combating epidemics/pandemics and specifically the coronavirus infection

	Strongly disagree	Disagree	Neutral	Agree	Strongly agree
The JPA has a role in preparing you to deal with epidemics/pandemics such as coronavirus.	24 (3.3%)	61 (8.4%)	199 (27.4%)	224 (30.9%)	218 (30.0%)
The JPA should be sending you awareness emails on the current coronavirus pandemic.	1 (0.1%)	11 (1.5%)	82 (11.3%)	266 (36.6%)	366 (50.5%)
The JPA should provide online educational workshops on the pandemic coronavirus.	3 (0.4%)	7 (1.0%)	89 (12.3%)	273 (37.6%)	354 (48.8%)
The JPA should monitor the availability of the medications used in the management of the coronavirus in pharmacies.	4 (0.6%)	6 (0.8%)	68 (9.4%)	238 (32.8%)	410 (56.4%)
The faculties of pharmacy and JPA should join forces to produce one educational module for the management of the coronavirus pandemic.	2 (0.3%)	4 (0.6%)	64 (8.8%)	240 (33.1%)	416 (57.3%)

### Results from the online focus group meeting

The focus group participants included seven policymakers (5 females and 2 males with professional experience over 5 years) including a dean’s assistant, a representative of a national pharmacy practice group, and four academics with teaching experience from four different faculties of pharmacy in Jordan (two public and two private universities), in addition to a representative from the JPA. It was evident that there was a within-group saturation of data. Key discussions were around the themes of (1) pandemic preparedness training needs, (2) role of policies and policymakers, and (3) managing feelings of vulnerability.

In *Theme 1*, participants reported the need for a national course on epidemics/pandemics that should be included in the curricular content and using the academic environment and infrastructure to train students on how to counsel patients during pandemics. For example, being able to explain to the patients about the “why” and “how to” maintain social distancing and maintaining the needed consultations while counselling or referring patients to the concerned authorities. Mental health awareness was mentioned to be a significant part of any course to be developed to prepare pharmacists for these difficult emergent roles. During pandemics, people go through a challenging mental situation, and being at the front line, pharmacists can capture patients in need of help and refer them accordingly to receive the proper help needed. However, many pharmacists are afraid of contracting the virus during their duties and feel pressured being expected to contribute to the pandemic standing in the front line-often this resulted in ethical practice dilemmas. Two examples were given, one about a pharmacist who saw a patient lying unconscious in front of his pharmacy; the pharmacist was too worried and did not approach the patient and simply closed the pharmacy. and after that the patient turned out to have had a hypoglycemic shock [22]. The second example was about a pharmacist who did not allow patients to enter the pharmacy and served them across a table positioned outside the door of the pharmacy [23]. Other important challenges mentioned here was the quarantine arrangements of working staff and the increased workload.

In *Theme 2*, as for the role of the JPA, the JPA implemented numerous activities to help in combating the spread of the virus, such as obtaining permits for the pharmacists to travel to and from their pharmacies. The JPA also helped in managing the availability of the stocks of medications and other needed supplies such as hand sanitizers, masks, and gloves. Participants stated that the duration of quarantine should be regulated, and related protocols and supplies are a must. Participants mentioned that hospital pharmacists should also be allowed to have a greater role during this pandemic. An important point about research during the pandemic was brought up as the JPA could help pharmacy researchers requesting to run research studies on the coronavirus pandemic in obtaining quick approvals to conduct their studies, whether in the labs or in hospitals. All the participants acknowledged the supportive actions taken by the Ministry of Health in combating the spread of the coronavirus, in addition to the actions taken by the different healthcare institutions in Jordan.

In *Theme 3*, the urgent need for emotional support for the public and pharmacists themselves was mentioned when further comments were elicited from the participants. In addition, it was discussed that many pharmacists were too afraid to deal with patients, suspecting they would contract the virus if the patient they were dealing with was infected.

## Discussion

There is no doubt that pharmacists should be incorporated into pandemic plans as frontline healthcare professionals considering the importance of their role in establishing and augmenting the communication with other healthcare professionals and roles in patient education and essential medicine supply [24]. Pharmacy organizations at national levels should clearly define and clarify the roles of pharmacists and advocate for legislations and policies that support pharmacy practice during pandemic eras [24]. This study has highlighted that most pharmacists, whether working or still studying, believed that tertiary pharmacy education providers and pharmacy professional bodies have a strong role in preparing pharmacists to deal with epidemics/pandemics, including the current coronavirus pandemic. The mixed-method design and robust sample size of this study enable a generalizable understanding, and though contextualized to Jordan and the current COVID-19 pandemic, the findings may be of relevance to pharmacy practice globally.

Healthcare providers face significant emotional and mental health challenges during critical incidences such as epidemics and pandemics or refugee crises [25]. Pharmacists in this study expressed concerns about their new roles, which needed to be adopted urgently. The expectation of pharmacists about contributing to the pandemic management and hiding their fear from contracting the virus while performing their roles were all additional stressors outlined by study participants. In addition, pharmacists fear infection transmission to their families while they have to work with sick patients [25]. Such concerns are expected to affect pharmacists’ mental health negatively, and professional support to cope with the running situation is needed [25]. Some of the challenges mentioned in the focus group phase of this study was the quarantine of pharmacists and working staff and the increased workload. Brooks et al. reviewed the psychological effect of quarantine due to infectious disease outbreaks, including SARS and the pandemic flu, H1N1 [26]. They reported that quarantine is associated with myriad stressors including the duration, frustration, financial load, boredom, and insufficient supplies [26]. Focus group participants confirmed important facts—that the duration of quarantine should be regulated to be “just enough” for transmission rate dampening and not longer than required and that the provision of applicable protocols and needed supplies is vital [27]. Supporting the healthcare provider can play a crucial role in relieving their stress and mental status, mainly by training sessions and peer groups support [28]. Our findings illustrated that the majority of responders agreed that pharmacists as a part of healthcare workers should receive such training sessions for mental health support and well-being.

All tertiary pharmacy education providers have a vital role in preparing pharmacy students as a workforce during the pandemic era [24]. Having a course/study unit embedded within the curriculum to teach pandemic-related topics was agreed upon by the majority of participants in both phases of the study (qualitative and quantitative) [24]. Both university/institute-entrenched curriculums, as well as professional development online lectures/webinars/workshops supported by links to updated evidence-based information/resources on the coronavirus pandemic management, were called for. Although many faculties did run online lectures and webinars about the pandemic, no dedicated unit of study or modular training full course on this topic exists at any of the faculties in Jordan. It may be proposed that deans of pharmacy faculties in Jordan should jointly deliberate to set up a national pandemic training module for pharmacists/pharmacy students. Such a course should include both theory and practical elements that cover key public health approaches to pandemics and potential roles for pharmacists. The topics should cover not only public health, health promotion, vaccine development, immunity profiles, testing, contact tracing, and health education, but should also include coverage for preparing individual/organizational plans (e.g., what can a pharmacy organization do if members of staff are infected, for example preparing back-up rosters) and communication skills to negotiate with patients potentially hoarding or those excessively worried. Such training and skills can help prepare a workforce competent in delivering essential services in a pandemic setting.

Along those lines, participants in the present study perceived the role of the JPA in the COVID-19 pandemic as important. The JPA was believed to have a vital role in different ways, such as by sending awareness emails, patient education materials, and online educational workshop provision. Advocacy roles for organizations such as the JPA were seen as particularly pertinent as many pharmacists felt unconnected and afraid of contracting COVID-19, believing that working in a pharmacy exposed them to the infection. The information elicited from the focus group meeting indicated that the JPA did not hold enough online lectures or webinars before or during the time this manuscript was prepared, a situation that can be improved for better response to professional needs in the future. Another key role for JPA was thought to involve the monitoring of the availability of the needed medications used in the management of the coronavirus pandemic; this role was well enacted in Jordan. All in all, most participants saw a need for the pharmacy education providers and the JPA to join forces to produce a combined, comprehensive training module that can serve as a reference source for updated information to pharmacists/pharmacy students during the coronavirus pandemic.

It has been said before, “Planning for a pandemic or other public health event does not happen at the time of the event. It needs to happen beforehand between different associations” [29] and “Pandemic planning allows health departments and pharmacies to coordinate efforts in advance of emergencies and ensure community resources are leveraged to their fullest extent” [29]. Hence, to ensure an effective role in the management of pandemics, pharmacists need prior training to be ready to deal with the emergency. The results of this study showed that half of the participants did not attend or attended only one continuous professional development (CPD) workshop during the previous year. Although none of the planned CPD events were around pandemic preparedness, with a lower rate of CPD workshop attendance, it is hard to ensure the readiness of pharmacists in combating epidemics and pandemics, such as the coronavirus pandemic, when they occur. When considering possible barriers preventing pharmacists from attending educational workshops, it is informative to look at their point of view. Lack of availability of educational workshops is a barrier preventing pharmacists from delivering their roles in different areas that have been documented previously [30]. These barriers need to be considered in making CPD options available; in fact, improved technological access for virtualized self-directed learning should be a priority, and a key observation, of course during the COVID-19 pandemic.

It is important for pharmacists and policymakers to be a part of the medical initiatives established in response to the COVID-19 pandemic. The JPA for example provided material support to the Ministry of Health helped in distributing six-thousand face masks for the benefit of Jordan’s sanitation workers [31], aided community pharmacists in obtaining permissions to open their pharmacies to serve their patients [32], and launched a service called “Hello Pharmacist” to help citizens identify the nearest pharmacy depending on their geographical location [33]. The Jordan Food and Drug Administration (JFDA) allowed community pharmacists and hospitals to provide free delivery of medications to patients’ homes once they registered in a database that was developed for this purpose to help patients obtain their medications easily and on time (delivering medications to patients’ homes is not allowed in Jordan under normal circumstances) [34]. Pharmacists referred patients showing symptoms of COVID-19 immediately to the hospitals in charge of COVID-19 patients under the supervision of the Ministry of Health [35]. Many pharmaceutical companies in Jordan provided free awareness webinars, highlighting the role of pharmacists during the pandemic [36]. Other services could be also trialed, such as teleconsultations, and Home Medication Management review services proved effective in other humanitarian settings, including the most current Syrian refugee crisis [37]. Policymakers can run certain pharmacy pandemic exercises to train pharmacists to distribute and dispense medications, such as antiviral drugs from public stockpiles [12] and administer vaccines during severe influenza pandemics which can prove effective [10]. In addition, data collected from the current handling of the coronavirus pandemic by pharmacies could be used by policymakers to plan and prepare for future pandemics.

This study comes with some limitations. With the methodology involving an online questionnaire, it meant that participants were not met face-to-face. In addition, the authenticity and reliability of the data obtained from the study “Facebook page” and through the “WhatsApp groups” (considered an official way of communication in Jordan and is officially recognized by the governmental bodies) could have been affected. However, incorporating such methodology was the only possible way to run the study during the current COVID-19 pandemic. Other alternative and authentic media could be used; reaching out to the participants on their professional email accounts, for example, could have been more authentic. However, during the pandemic, anecdotal comments from researchers indicated that people were not frequently checking their emails. Representativeness in the profile about participants is a limitation considering that most participants were from Amman, the capital, where the JPA is located and most healthcare facilities. The survey is not validated but the items were put together in real time, based on current literature, and reviewed by an expert team of clinical pharmacists and academics.

## Conclusions

This study is the first to highlight the main barrier facing many pharmacists and pharmacy students, namely the emotional and mental challenges they have to face during the current COVID-19 pandemic. Pharmacists in this study expressed concerns about their new urgent roles and know at the same time that they are expected to contribute to the pandemic management. Pharmacists and pharmacy students believed that pharmacy education providers have a key role in preparing them to deal with epidemics/pandemics including the coronavirus pandemic. The majority believed that a modular course or curriculum-embedded training on epidemic/pandemic management is sorely needed and that their alma mater faculties should provide them with online lectures/webinars and online information resources during such circumstances. The case was similar for their perception of the role of the JPA, and the majority believed the JPA should be sending them awareness emails and provide online educational workshops on the pandemic. Finally, participants believed that pharmacy education providers and the pharmaceutical associations should join forces to produce one educational module for the management of the coronavirus pandemic. Results of this study have important international applicability, as pharmacists all over the world share similar fears while being obliged to perform their responsibilities and engage with public society. Exploring and executing interventions to overcome this vital issue is the responsibility of not only local but international pharmaceutical associations. In addition, countries worldwide share similar systems when it comes to pharmacy education and post-graduation training and practice, hence pharmacists’ perspectives reported here can be of benefit to any healthcare system around the world [38].

## Data Availability

The data will be made available by the corresponding author upon request.
